# Genetic Diversity, QTL Mapping, and Marker-Assisted Selection Technology in Cotton (*Gossypium* spp.)

**DOI:** 10.3389/fpls.2021.779386

**Published:** 2021-12-16

**Authors:** Fakhriddin N. Kushanov, Ozod S. Turaev, Dilrabo K. Ernazarova, Bunyod M. Gapparov, Barno B. Oripova, Mukhlisa K. Kudratova, Feruza U. Rafieva, Kuvandik K. Khalikov, Doston Sh. Erjigitov, Mukhammad T. Khidirov, Madina D. Kholova, Naim N. Khusenov, Roza S. Amanboyeva, Sukumar Saha, John Z. Yu, Ibrokhim Y. Abdurakhmonov

**Affiliations:** ^1^Institute of Genetics and Plant Experimental Biology, Academy of Sciences of the Republic of Uzbekistan, Tashkent, Uzbekistan; ^2^Department of Biology, National University of Uzbekistan, Tashkent, Uzbekistan; ^3^Center of Genomics and Bioinformatics, Academy of Sciences of the Republic of Uzbekistan, Tashkent, Uzbekistan; ^4^Crop Science Research Laboratory, USDA-ARS, Washington, DC, United States; ^5^Southern Plains Agricultural Research Center, USDA-ARS, Washington, DC, United States

**Keywords:** cotton, genetic diversity, DNA markers, QTL mapping, GWAS, marker-assisted selection

## Abstract

Cotton genetic resources contain diverse economically important traits that can be used widely in breeding approaches to create of high-yielding elite cultivars with superior fiber quality and adapted to biotic and abiotic stresses. Nevertheless, the creation of new cultivars using conventional breeding methods is limited by the cost and proved to be time consuming process, also requires a space to make field observations and measurements. Decoding genomes of cotton species greatly facilitated generating large-scale high-throughput DNA markers and identification of QTLs that allows confirmation of candidate genes, and use them in marker-assisted selection (MAS)-based breeding programs. With the advances of quantitative trait loci (QTL) mapping and genome-wide-association study approaches, DNA markers associated with valuable traits significantly accelerate breeding processes by replacing the selection with a phenotype to the selection at the DNA or gene level. In this review, we discuss the evolution and genetic diversity of cotton *Gossypium* genus, molecular markers and their types, genetic mapping and QTL analysis, application, and perspectives of MAS-based approaches in cotton breeding.

## Introduction

Cotton is one of the oldest cultivated crop plants and it is grown as the main source of raw materials for the textile industry. More than 103 million tons of textile fibers were consumed in 2019 and cotton fiber had a market share of approximately 24% in 2020 over 26 million tons of cotton was produced worldwide (ICAC, 2021). Cotton is valued for its fiber quality in the global market and it determines price of the fiber. Cotton fiber faces a grave challenge by a chemically produced (synthetic) fiber. Synthetic fibers currently control over 75% of the global market share in textile fiber consumption (ICAC, 2021). Therefore, competition from synthetics has increased textile industry demands for cotton fiber with high quality and superior spinning performance. However, one of the serious impediments to improve the cotton fibers is the narrow genetic base in Upland cotton. In this regard, there is a constant need to introduce genetic diversity into the new varieties with excellent fiber quality and high yield potentials (Abdurakhmonov et al., 2008, 2009; Yu et al., 2014).

Creating new varieties using traditional breeding methods, specifically, the introduction of genes of desirable traits to the elite cotton from a donor source to the elite is very laborious and requires at least 10 years of hard work (Abdurakhmonov et al., 2009). At the same time, there is often a negative correlation between desired traits, such as fiber quality and fiber yield or resistance to abiotic environmental stress factors, which often prevent the breeder from the effective selection and breeding (Griffith and Crowfoot, 1934; Nicholson, 1960; Muthukumaran, 2016).

Similar problems can be solved by introducing modern biotechnological approaches based on the use of molecular markers in breeding programs (Abdurakhmonov et al., 2008, 2009). Modern breeding programs for the accelerated generation of new varieties provide in-depth study of breeding material at both the phenotypic and genotypic levels. DNA markers are commonly referred to as molecular markers, although previously widely used isozymes and other marker systems were based on protein polymorphism. With the introduction of DNA marker technology into the practice of plant breeding, new opportunities have emerged for studying genetic diversity, identifying and improving economically useful crop traits (Preetha and Raveendren, 2008). The advent of molecular marker technology provides breeders with powerful new tools for identifying complex quantitative traits. Moreover, DNA marker technology allows breeders to increase efficiency and reduce costs and time to create new varieties and hybrids compared to traditional breeding methods. A large number of DNA markers and genes controlling resistance to biotic and abiotic stresses, yield, and quality traits have been identified and mapped for many crop species in recent years (Zhang et al., 2013; Han et al., 2019; Wang et al., 2019).

Molecular markers gave a great opportunity to improve the efficiency and precision of crop improvement programs *via* marker-assisted selection (Collard and Mackill, 2008). The use of DNA markers in plant breeding is called marker-assisted selection (MAS) and it is a component of the molecular breeding approach (Collard and Mackill, 2008). MAS technology allows conducting the selection at any stage of plant growth and development. In short, the development of the MAS technology aimed at the selection of crops led to high achievements in genomics, which became a vital part of agricultural science.

## Taxonomy, Evolution, And Genetic Diversity Of Cotton *Gossypium* Genus

The *Gossypium* L. (the cotton genus) has a long history of taxonomic and evolutionary study. The *Gossypium* genus, belonging to the tribe *Hibisceae* (*Malvaceae* family), includes approximately 46 diploid and 7 allotetraploid species (Fryxell, 1979; Wendel and Albert, 1992; Wendel et al., 2009; Paterson et al., 2012; Wendel and Grover, 2015). The diploid (*n* = x = 13) species of cotton is classified into eight (A-G and K), and the tetraploid (*n* = 2x = 26) species into one (AD) cytogenetic group (Brubaker et al., 1999; Wendel and Cronn, 2003). Mainly 4 species are cultivated in around 90 cotton producing countries of the world: *G. hirsutum* L., which occupies more than 90% of the total area, followed by *Gossypium barbadense* L., approximately 8% and only 1% two diploid species – *G. arboreum* L. and *G. herbaceum* L. The genome size of diploid cottons varies from about 880 to 2,500 Mb, as well as the tetraploid cotton genome has an estimated size of 3,000 Mb (Hendrix and Stewart, 2005).

Based on their origin, diploid cotton species are divided into two types: African-Asians and Australian (Blenda et al., 2012). *G. arboreum* L. and *G. herbaceum* L, having twisted fiber, were originally grown on the Asian continent. Subsequently, as a result of hybridization between diploid A-genomic (Asian) and D-genomic (Mexican) representatives, which occurred about 1.5 million years ago, formed five allotetraploid species of cotton (Yu et al., 2013).

Upland cotton (*G. hirsutum*) is widely cultivated, industrial cotton among all species of the *Gossypium* genus (Iqbal et al., 2001). The origin of this species is considered Guatemala, but it is distributed throughout Central America and in Caribbean countries. According to Mauer (1954), there are four groups of subspecies of *G. hirsutum*: *mexicanum, punctatum, paniculatum, and euhirsutum*. These four groups of subspecies include several wild races, such as *yucatanense*, *richmondi*, *latifolium*, *palmeri*, *morilli*, *purpurascens*, and their variety samples, as well as a number of cultivated samples (Adams et al., 2004).

*Gossypium barbadense* (Egyptian, Sea Island or Pima cotton) is widely distributed throughout most of South America, Southern Mesoamerica, and the Caribbean (Rathore et al., 2006). This species of *Gossypium* genus initially sprouted on ribbed coastal islands and in the valleys of the United States and was named Sea Island cotton. Then, Sea Island cotton was introduced into the Nile Valley of Egypt, where it is widely cultivated for the production of long and thin fibers (Abdalla et al., 2001).

The remaining three tetraploid species (AD_3_–AD_5_) are common in other regions. So, for example, *G. mustelinum* Miers ex Watt widely distributed in Northeast Brazil (Wendel et al., 1994), *G. darwinii* Watt is an endemic of the Galapagos Islands (Wendel and Percy, 1990), and *G. tomentosum* Nutt ex Seem is endemic to the Hawaiian Islands (Hawkins et al., 2005). All of them are truly wild species (Westengen et al., 2005). Genetic diversity represents the existence of various variants of biological forms or the degree of morphological and physiological features of organisms within populations (often called traits), which are essential for biological individuals both for a positive response to a rapid change in the environment and for their survival. The lack of genetic diversity or its narrowness in various types of crops creates the potential threat to plant productivity due to the vulnerability of genetically homogeneous varieties to new biotic and abiotic stresses. Consequently, the broad genetic diversity of crops has the potential to protect them from new diseases, pests, and unexpected global environmental changes (Abdurakhmonov et al., 2007).

Thus, the genus *Gossypium*, covering large geographical and ecological niches, has a wide amplitude of morpho-biological and genetic diversity, preserved in centers of origin of cotton *in situ*, in collections of germplasm of cotton *ex situ*, as well as in materials of breeding programs throughout the world. These resources can be successfully used in cotton breeding programs to transfer economically valuable traits from wild species to the cultivated genotypes in order to create promising competitive varieties.

## Importance of Genetic Diversity For Cotton Improvement

Cotton faces various problems in production and marketing, such as competition from synthetic fiber, wide variability from year to year in yield, and plus new requirements for fiber quality due to technological changes in the textile industry (Perkins et al., 1984; Esbroeck et al., 1999). A longer fiber, like that of low-yielding cotton species *G. barbadense*, is genetically stronger, thinner, and more uniform than a shorter fiber of the widely sown, early-growing and high-yielding cotton *G. hirsutum* (Perkins et al., 1984). Changing these fiber properties in medium fiber cotton is a big challenge facing cotton breeding programs around the world (Esbroeck et al., 1999).

Since *G. hirsutum* is the most widely cultivated species in the world, due to its high yield, early maturity, and unpretentiousness of cultivation, much research has been devoted to the analysis of its genetic diversity (Abdurakhmonov et al., 2008, 2009; Blenda et al., 2012). At the same time, less attention is paid to the study of *G. barbadense*, the second most cultivated cotton species, since the varieties of this species have lower yields and weaker indicators of other economically important traits compared to the *G. hirsutum* species (Wendel and Percy, 1990; Fryxell, 1992; Yu et al., 2013). Cotton researchers constantly carry out selection measures for crossing these two species, with the goal of transferring the superior fiber quality components specific only to *G. barbadense*, to the cultivated varieties of *G. hirsutum* (Fryxell, 1992). It should be noted through interspecific hybridization between *G. barbadense* and *G. hirsutum* species, the desired alleles for most QTLs associated with fiber quality are transmitted from *G. barbadense* (Lacape et al., 2005). Moreover, scientists proved that *G. hirsutum*, in turn, can also contribute to the improvement of fiber length, strength, and micronaire (Lacape et al., 2005; Yu et al., 2013). This discovery confirms the assumption that in generations of interspecific hybrids having a mosaic genome, best gene allele combinations can be achieved (Tanksley and Nelson, 1996). In most cases, the use of interspecific crossing of *G. barbadense* with *G. hirsutum* with classical breeding methods to improve fiber quality traits, such as length, strength, and micronaire in Upland cotton, did not lead to the expected stable introgression. According to the study published by the researchers (Tanksley and Nelson, 1996; Lacape et al., 2005; Yu et al., 2013, 2014), the solution to these problems at the genetic level requires knowledge of broader variation in the cotton germplasm. However, according to the review by Zhang et al. (2014), new introgression lines with high yields and fiber quality were developed as a result of interspecific hybridization between *G. hirsutum* and *G. barbadense* (Zhang, 2011).

Narrow genetic diversity can be caused by the intensive use of one or several closely related genotypes in breeding programs (Iqbal et al., 2001) or the consequence of a “genetic bottleneck” at the historical transformation of wild plants into cultural forms, which led to the subsequent distribution of a limited number of genotypes (Abdurakhmonov et al., 2007). The productivity, viability, and success of cotton breeding, like many other crops, also depend on the diversity of the gene pool (Esbroeck et al., 1999). According to the authors (Iqbal et al., 2001), the existing and projected problems of the world cotton breeding programs related to the narrowness of the genetic base of the germplasm arise because of the complexity of the tasks and the lack of new genomic approaches for mobilizing beneficial genetic variations from various exotic cotton species of the *Gossypium* genus into breeding varieties.

## Molecular Markers and Their Types

**Figure 1:**
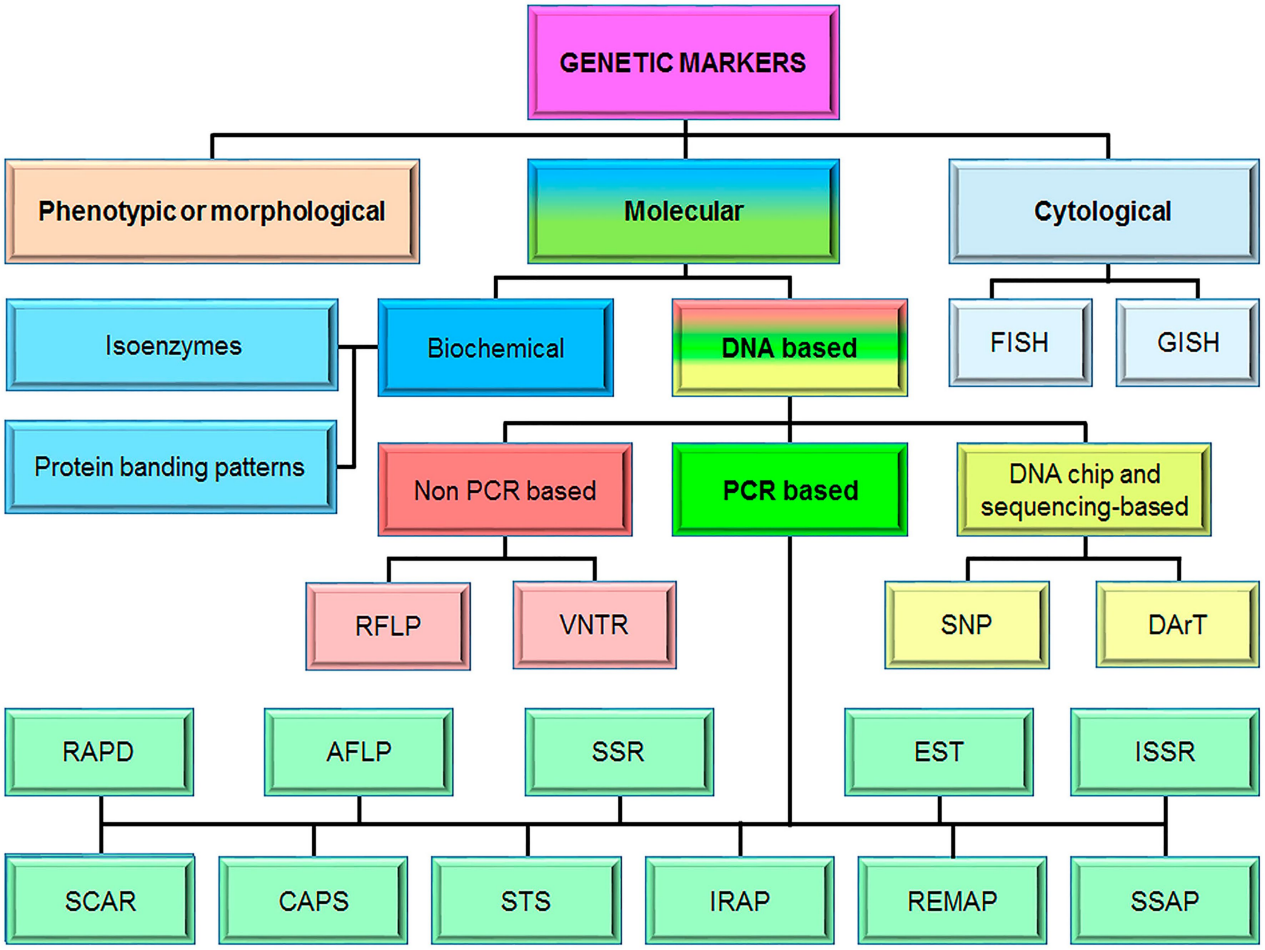
The classification of genetic markers. FISH, fluorescence *in situ* hybridization; GISH, genome *in situ* hybridization, RFLP, restriction fragment length polymorphism; VNTR, variable number tandem repeat; SNP, single-nucleotide polymorphism; DArT, diversity arrays technology; RAPD, random amplified polymorphic DNA; AFLP, amplified fragment length polymorphism; SSRs, simple-sequence repeats; EST, expressed sequence tag; ISSR, inter simple-sequence repeat; SCAR, sequence-characterized amplified region; CAPS, cleaved amplified polymorphic sequences; STS, sequence-tagged sites; IRAP, inter-retrotransposon amplified polymorphism; REMAP, retrotransposon-microsatellite amplified polymorphism, and SSAPs, sequence-specific amplification polymorphisms.

Genetic markers are any observable inherited traits that differ individually from one another. For many years, they have been used to characterize genetic diversity for crop improvement. This tool is particularly useful in analyzing complex quantitative traits. Genetic markers can be divided into three main types: morphological (or phenotypic), cytological, and molecular markers (Figure 1). Morphological markers represent the actual polymorphism of the phenotype and they are identified easily, quickly, and most importantly with minimum laboratory equipment (Nadeem et al., 2018). The physical maps based on morphological and cytological markers lay a foundation for genetic linkage analysis using molecular methods (Jiang, 2013). However, direct use of cytological markers has been very limited in genetic mapping and plant breeding (Jiang, 2013; Ahmad et al., 2017; Nadeem et al., 2018). Nevertheless, the number of informative morphological markers is very small (Eagles et al., 2001). The low occurrence rate and many other deficiencies did not allow morphological markers to enter widely into selection practice.

Until recent advances in molecular genetics, breeders have improved both qualitative and quantitative hereditary traits by traditional breeding methods based on evaluation and selection for phenotypic variation, which are resource-intensive (Said et al., 2015a). Currently, two main types of molecular markers, biochemical and DNA markers are available for genetic studies (Lander and Botstein, 1989; Jansen, 1994). It should be noted that the first molecular markers were created based on the analysis of protein polymorphism. However, the possibilities of biochemical markers are limited by the low level of protein polymorphism in populations, restrictions in the choice of biological material, and the time of its collection (Kumar et al., 2009).

DNA-based molecular markers are genetic markers that are analyzed at the DNA level. This marker system plays a huge role in the study of gene inheritance and their allelic status. Such markers are used to analyze genetic polymorphism and phylogenetic relationships between species, populations, and individuals, as well as to identify diagnostic markers that are closely linked to the genes controlling the economically valuable traits of crops (van Ooijen, 1992). An important tool for finding DNA markers is linkage mapping, which allows to combine phenotypic data and DNA polymorphism data. Currently, there are many different types of DNA markers, and their numbers are constantly increasing with the achievement of modern technologies and knowledge of individual genes and genomes of plants in general. So, DNA markers can be divided into three main groups: markers based on hybridization (or non-PCR based), markers based on PCR, and markers based on DNA chips (Singh and Singh, 2015). The PCR-based marker system is very popular and more widely utilized among these groups of DNA markers (Reiter, 2001). Moreover, it is PCR-based DNA markers that are widely introduced into the plant selection process. One of the most informative types of PCR-based DNA markers is microsatellite or SSR (simple-sequence repeat) markers, as they are widely distributed in the genome and have a high level of polymorphism (Vieira et al., 2016).

Thus, molecular or DNA markers are genetic tools that allow plant breeders to perform a variety of tasks. Especially, DNA markers play an important role in the study of genetic polymorphism, inheritance of genes, and their allelic state, in phylogenetic analysis, as well as identification of QTLs that are closely linked with genes controlling the economically valuable traits of plants (Bruford et al., 2003; Mittal and Dubey, 2009).

## Genetic Mapping and Qtl Analysis For Agronomically and Economically Valuable Traits in Cotton

### Description of Genetic Mapping Approaches

One of the main tasks of molecular markers is the mapping of genes and quantitative trait loci (QTLs; Jansen, 1993). The theory of QTL mapping was first described by Karl Sax (1923) when he observed segregation of seed weight associated with segregation for a seed coat color marker in *Phaseolus vulgaris* L. (Sax, 1923). He noted that one gene controlling seed color should be associated with one or more polygenes controlling seed size. Since the development of the first molecular markers (Grodzicker et al., 1974), a large amount of theoretical and practical results and methodological developments describing the stages of molecular genetic mapping have been accumulated (Lander and Botstein, 1989; van Ooijen, 1992; Jansen, 1994). The concept of genetic linkage mapping is based on the study of the genome of an organism by DNA markers (Goldstein and Weale, 2001; Jannink and Walsh, 2002; Oraguzie et al., 2007), determining the relative position of these markers on linkage groups and determining their genetic association with QTLs (van Ooijen, 1992). Genetic mapping is mainly accomplished in two ways; the first, traditional method, the so-called linkage analysis or QTL mapping (Jansen, 1994) has already become a classic method. This mapping study is carried out using experimental (biparental) populations of F_n_ generations (Lewis, 2002), backcross (BC_n_), recombinant inbred lines (RIL), and/or doubled haploid lines (DHL).

The second method used in the construction of modern genetic maps of plants is the analysis of linkage disequilibrium (LD) and association mapping (Goldstein and Weale, 2001; Jannink and Walsh, 2002). LD mapping uses different lines from natural populations or germplasm collections (Asíns, 2002; Glazier et al., 2002). Thus, when mapping, individuals are divided into genetic classes for each DNA marker used (van Ooijen, 1992). Next, the values and variations of the parameters are calculated and compared between the classes. The identified polymorphism between genetic classes provides information about the relationship of the marker used with the phenotype of interest, and its connection with the QTL (Xu et al., 2017a). After detecting genes those regulate quantitative traits, positional mapping is used based on statistical data analysis.

Currently, a rich arsenal of QTL-mapping methods has been created, which implements various approaches (Wan et al., 2009). The developed methods are based on the well-known principles of parametric and nonparametric analysis of linkage, as well as new approaches using the analysis of components of dispersion (Zhang and Gai, 2008), analysis of associations (Jia et al., 2014), and multipoint mapping (Liu and Muse, 2005). The development of statistical methods follows the path of increasing their power and stability of methods to inaccuracies of genetic models and the incompleteness of empirical data (Price, 2006).

### QTL Analysis for Fiber Quality, Stress and Disease Resistance, and Some Morphological Traits in Cotton

Since the development of molecular mapping technology, researchers have created hundreds of genetic maps and identified many QTLs associated with economically valuable traits (Saranga et al., 2001; Gao et al., 2003; Du et al., 2004; Abdurakhmonov et al., 2005; Bolek et al., 2005; Lacape et al., 2005; Yin et al., 2006; Abdurakhmonov et al., 2007; Wang et al., 2007; Qin and Zhang, 2008; Yang et al., 2008; Zan et al., 2008; Goicoechea et al., 2009; Jiang et al., 2009; Wan et al., 2009; Said et al., 2013, 2015a; Tang et al., 2014; Zhang et al., 2015; Kushanov et al., 2016; Diouf et al., 2018; Zhao et al., 2018). As a primary goal, the cotton research community has set QTL mapping with molecular markers associated with fiber yield, quality, and yield traits (Wang et al., 2007; Abdurakhmonov et al., 2008; Abdurakhmonov et al., 2009; Said et al., 2013; Yu et al., 2013; Tang et al., 2014). Some QTLs related to environmental stress resistance, such as drought (Saranga et al., 2001), as well as loci associated with the formation and morphology of stems and leaves (Said et al., 2013), chlorophyll content (Qin and Zhang, 2008), natural leaf defoliation (Abdurakhmonov et al., 2005), and fertility restoration genes (Zhao et al., 2018), are also mapped. Currently, 4,892 QTLs identified either in the populations of *G. hirsutum* or *G. hirsutum* × *G. barbadense* and presented in 156 publications are available in the Cotton QTLdb database^1^ (Said et al., 2015b).

The QTL analysis of fiber quality traits of cotton began about 25–30 years ago (Kloth, 1993, 1995). Today, single-nucleotide polymorphism (SNP) markers-based genome-wide association study (GWAS) is widely used for identifying genomic regions that attending to control economically important traits in both natural and experimental populations of cotton. Gapare et al. (2017) have conducted GWAS on natural cotton populations to identify genetic contributions to the fiber quality, plant architecture, and stomatal conductance traits (Gapare et al., 2017). They have used Illumina CottonSNP63 K SNP array for genotyping. The results of analysis showed that 17 and 50 significant SNPs associated for fiber length and micronaire, respectively. Sun et al. (2018) performed a GWAS of fiber quality traits of 719 diverse accessions of upland cotton using Cotton 63 K Illumina Infinium SNP array (Sun et al., 2018). Germplasm resources were screened using more than 10.5 thousand polymorphic SNPs distributed in 26 chromosomes, and 46 significant SNP markers related to fiber quality traits were identified. These important SNPs are distributed on 15 chromosomes and are involved in 612 unique candidate genes, many of which are associated with polysaccharide biosynthesis, signal transmission, and protein translocation. In addition, scientists have identified 163 and 120 fiber genes related to length and strength, respectively. Ma et al. (2018) have identified more than 3.6 million SNPs by re-sequencing 419 cotton accessions and conducted GWAS of 13 fiber-related traits (Ma et al., 2018). More than 7.3 thousand SNPs were associated with fiber quality traits and covered 4,820 genes; more fiber-related genes were determined in D subgenome than in the A subgenome. Liu et al. (2020) have identified 42 SNPs and 31 QTLs significantly associated with five fiber quality traits (Liu et al., 2020). Twenty-five QTLs are the same as QTLs identified in previous studies, and six novel QTLs were firstly identified in their work. In these QTL regions, 822 genes were determined as well two pleiotropic SNPs associated with fiber elongation, strength, length, uniformity, and strength were identified.

Besides, Fang et al. (2014) have identified 131 fiber QTLs and 37 QTL clusters on experimental mapping population using 2,132 polymorphic SSR markers out of 15,538 SSRs (Fang et al., 2014). Two QTL clusters were determined on chromosomes 7 and 16. Comparison of 131 QTLs showed that 77 were identified in the previously studies, and 54 novel QTLs. Recently, Islam et al. (2016) used an Upland cotton multi-parent advanced generation inter-cross (MAGIC) population, developed through random mating of 11 diverse cultivars for five generations, in a molecular map of SNP markers associated with fiber traits from four environments (Islam et al., 2016). They used a high-throughput genotyping approach of Genotyping-by-Sequencing (GBS) developing about 6,071 SNP markers and 223 microsatellite markers of 547 recombinant inbred lines (RILs) of the MAGIC population. They used a GWAS using a mixed linear model to identify markers significantly associated with fiber QTLs. They discovered one QTL cluster associated with four fiber quality traits [short fiber content (SFC), strength (STR), length (UHM), and uniformity (UI)] on chromosome A07. They further identified several candidate genes related to fiber quality attributes in this region. Gene expression and amino acid substitution analysis suggested that regeneration of bulb biogenesis 1 (GhRBB1_ A07) gene is a candidate for superior fiber quality in Upland cotton. The DNA marker CFBid0004 designed from an 18 bp deletion in the coding sequence of GhRBB1_A07 in Acala Ultima is associated with the improved fiber quality in the MAGIC RILs and 105 additional commercial Upland cotton cultivars. Using GBS technology and a MAGIC population enabled more precise fiber QTL mapping in Upland cotton. Normally Acala Upland cotton lines carried superior fiber quality traits compared to other Upland cotton. Thyssen et al. (2018) have identified identify seven highly significant fiber quality loci associated with six major cotton fiber quality traits in a MAGIC population using GWAS and whole-genome sequencing (Thyssen et al., 2018). At these loci, they found 14 genes with non-synonymous SNPs. Sarfraz et al. (2021) have conducted subsequent genome-wide predictions along with association analyses that uncovered a set of highly significant key SNPs related to agronomic and fiber quality traits (Sarfraz et al., 2021). The integration of a GWAS with RNA-sequence analysis yielded 275 candidate genes near the key SNPs. The main part of candidate genes is associated with fiber micronaire and lint percentage. As well, 54 putative candidate genes were identified in association with the heterosis of quoted traits.

Xu et al. (2020) have carried out the study to identify candidate genes related to fiber quality traits through the integration of meta-QTL, significant SNP, and transcriptomic data (Xu et al., 2020). Scientists have used fiber quality traits associated 884 QTLs from 12 studies for meta-QTL analysis based on reference genome TM-1. As a result of meta-analysis, 74 meta-QTLs were identified, in particular 19 meta-QTLs for fiber length, 18 meta-QTLs for fiber strength, 11 meta-QTLs for fiber uniformity, 11 meta-QTLs for fiber elongation, and 15 meta-QTLs for micronaire. As well as with 8,589 significant SNPs associated with fiber quality traits gathered from 15 studies, 297 candidate genes were determined in the meta-QTL intervals, 20 of which showed high expression levels specifically in the developing fibers.

Cotton is mainly grown in the regions which are affected by abiotic stresses, such as drought and salt (Abdelraheem et al., 2019). There is an urgent need to study of genetic bases of abiotic stress resistance and to improve drought resistance of cotton (Li et al., 2020c). Recently, Abdelraheem et al. (2019, 2021) have identified drought and salinity stress resistance-related QTLs using SNP markers on an inter-cross mapping population (Abdelraheem et al., 2019, 2021). A total of 20 QTL were determined for drought tolerance and 23 QTL for salt tolerance out of 473,516 polymorphic SNPs. Nine QTL identified were in common between drought and salt tolerance, indicating a general genetic basis for both traits. Li et al. (2020a) have studied the genetic architecture for drought resistance in cotton using phenomics-based GWAS analysis (Li et al., 2020a). In their study, scientists have used an automatic phenotyping platform to examine drought stress tolerance at the seedling stage, across a natural population of upland cotton accessions. The phenomics data allowed to identify 390 genetic loci and drought tolerance-related genes by GWAS. Zhu et al. (2020) have conducted GWAS using 57,413 high-quality SNPs in 316 *G. hirsutum* accessions that grown under four salt conditions over 2 years and identified a total of 42, 91 and 25 stable QTLs for single boll weight, lint percentage, and boll number per plant, respectively (Zhu et al., 2020).

At the same time, great progress was achieved in the QTL mapping, determining resistance to Verticillium wilt (VW) and Fusarium wilt (FW). Most studies on the mapping of resistance to this pathogen have been conducted in germplasm accessions and diverse mapping populations. For instance, Li et al. (2017) have conducted a study to examine the genetic architecture of cotton Verticillium wilt disease resistance (Li et al., 2017). They performed a GWAS in 299 cotton accessions and 85,630 SNPs detected using the specific-locus amplified fragment sequencing (SLAF-seq) approach and were detected a total of 17 significant SNPs. Haplotype block structure analysis predicted 22 candidate genes for VW resistance. Zhang et al. (2019) have carried out GWAS analysis using 473,516 SNPs/Indels in 550 recombinant inbred lines (RILs) of multi-parent advanced generation inter-cross (MAGIC) population (Zhang et al., 2019). Consequently, a significant QTL for FW resistance on chromosome c14 was identified. An interesting aspect is that a major resistance gene (B12) for bacterial blight resistance and one QTL for Verticillium wilt resistance were also identified within the QTL region in this MAGIC population. Another group of scientists has conducted a GWAS using high-density genotyping with the CottonSNP63K array and identified a total of 15 and 13 QTL for VW and FW resistances were anchored by 30 and 56 significant SNP markers, respectively (Abdelraheem et al., 2020). Similar studies were also conducted by Bardak et al. (2021) to discover the genetic markers associated with the Verticillium wilt disease in a Worldwide Collection of Cotton (*Gossypium hirsutum* L.; Bardak et al., 2021). Through the association mapping analysis, common SNP markers were obtained using 4,730 SNP alleles. As a result, 23 markers were associated with defoliating (PYDV6 isolate) pathotype, 21 markers with non-defoliating (Vd11 isolate) pathotype, 10 QTL with Disease Severity Index (DSI) of the leaves at the 50–60% boll opening period, and 8 markers were associated with DSI in the stem section.

Also, some reports have been published on QTL and/or gene mapping of flowering time using diverse marker technology. Researchers have identified more than 30 candidate genes that are involved in various flowering processes in Upland cotton (Lai et al., 2011). In addition, Zhu and Kuraparthy (2014) managed to localize the photoperiod-sensitive locus Gb_Ppd1 and several closely related SSR markers on the cotton chromosome 25. Guo et al. (2008) presented the mapping of flowering-time QTL, assessed by node of first fruiting branches in cotton (Guo et al., 2008). Using more than four thousand SSR markers, researchers identified about 60 loci, associated with early maturing traits of cotton (Li et al., 2013b). Recently, using 212 SSR and 3 cleaved amplified polymorphic sequence (CAPS) markers, 6 QTLs were identified that directly associated with flowering time and photoperiodic flowering in the F_2_ population, whereas 7 QTLs were discovered in F_3_ generation (Kushanov et al., 2017). Li et al. (2020b) have reported a cotton genome variation map that is generated by the re-sequencing of 436 cotton accessions (Li et al., 2020b). Whole-genome scans for sweep regions identified 357 putative selection sweeps covering 112 Mb of the upland cotton genome, containing 5,184 genes. These genes were functionally associated with flowering-time control, hormone catabolism, aging, and defense response adaptations to climate changes.

Some QTLs related to the formation and morphology of stems and leaves (Said et al., 2013), chlorophyll content (Qin and Zhang, 2008), natural leaf defoliation (Abdurakhmonov et al., 2005), and fertility restoration genes (Zhao et al., 2018) are also mapped.

Although traditional QTL mapping based on the biparental crossing is still an important method for identifying desired genes/loci in plant chromosomes, it has nevertheless become a kind of “modern classical analysis” method. The disadvantage of this method is its low resolution only allowing for the evaluation of a few alleles over a rather long period of analysis. At the same time, markers detected during QTL mapping and specific for some lines may not be specific for other populations or germplasm of a given cultivation.

At the same time, another problem remains topical – to obtain genotypes that are not only resistant to diseases, but at the same time having high yield and superior fiber quality. One of the ways to solve this problem is the interspecific crossing between *G. barbadense* and *G. hirsutum* varieties, which differ in the indicated characteristics. However, these attempts are not always successful due to the problems associated with sterility, cytological impairments, and distorted segregation arising from interspecific crosses. Thus, QTL mapping is one of the powerful methods for improving agricultural crops, which allows using the marker-assisted selection technology to introgress the genes of interest from donor lines to breeding material.

## Application and Perspectives of Mas in Cotton Breeding

### Application of MAS

The concept of using linked genes has arisen to follow the inheritance of genes that control other traits. It was launched in 1961 by Thoday, who made the first attempt to map and characterize all polygenes that affect the line using monogenic markers (Thoday, 1961; Mutschler et al., 1987). When he worked with morphological markers, the main practical limitation was availability of few suitable markers are available.

**Figure 2:**
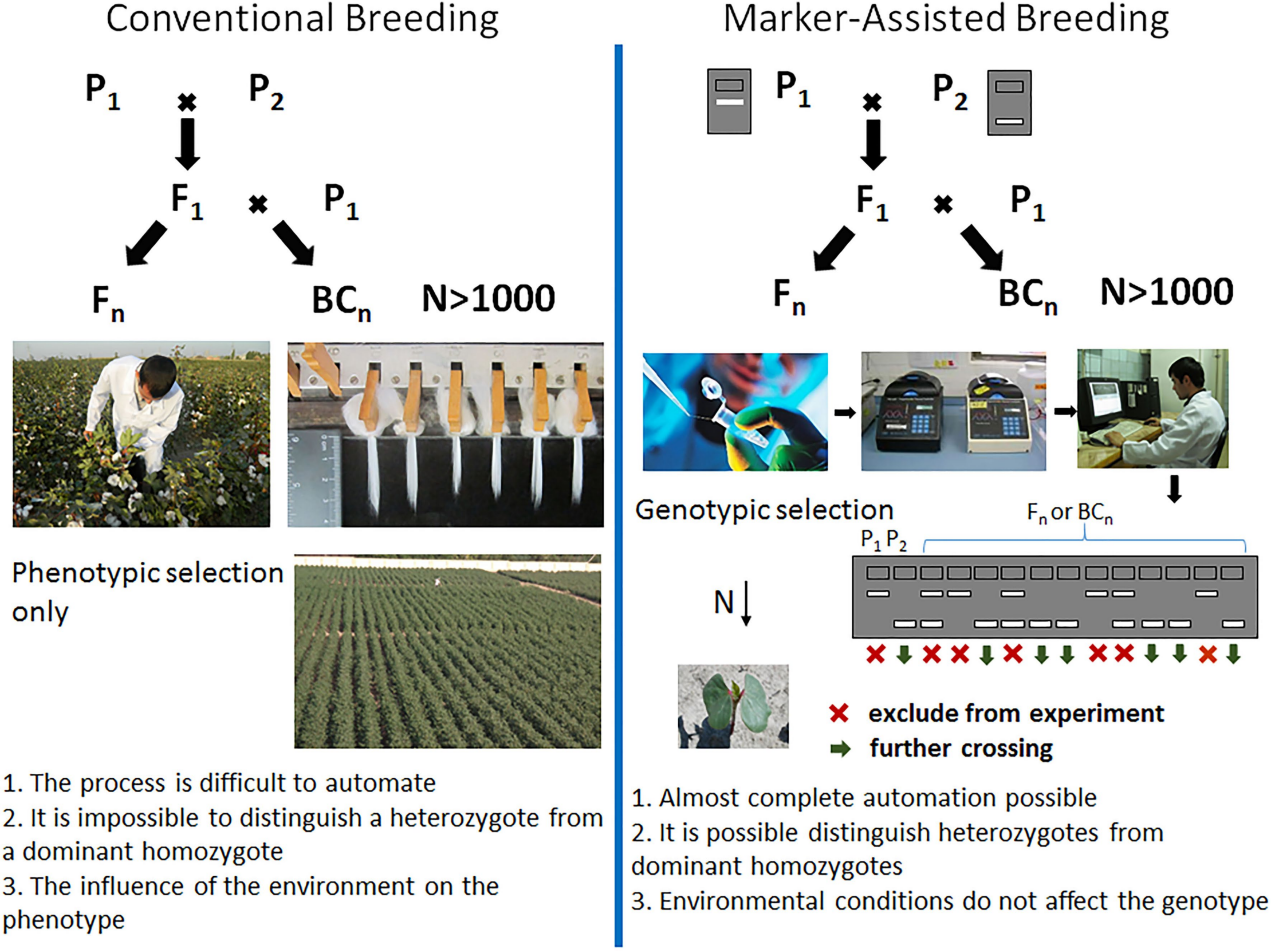
Marker-assisted selection in comparison with conventional breeding. P_1_ and P_2_ – parental genotypes, F_1_ – first generation hybrid, F_n_ – hybrid progeny obtained from first generation by self-pollination, and BC_n_ – backcross generations.

With the advent of DNA marker technology and the QTL-mapping approach, the possibilities of breeding for crop improvement have increased significantly. The use of marker-assisted breeding revolutionized the process of creating crop varieties, reducing field trials at an early stage of breeding, and reducing the time required by almost half (Figure 2). At the same time, DNA markers associated with traits of interest allow breeders to accurately select individuals based on genotype. This approach is very useful in cases where the trait of interest is complex and time consuming to assess. Moreover, the desired alleles in wild relatives with a low phenotype can also be identified with DNA markers. Such transgressive loci of wild species can be selected and used to create new varieties with a more desirable phenotype, introducing useful variations in crops.

To carry out marker-assisted selection, a large number of polymorphic markers must be identified by analyzing the whole genome. It is necessary to evaluate the marker informativity between the parents used in the crossing, and this can be used to assess the segregating population for the absence or presence of this genetic marker. The benefits of genetic selection can be maximized by increasing the genetic pool or population size so that individuals with an unusual genotype can be identified. At the same time, an increase in the number of markers used proportionally increases the reliability to assess the genome structure. In order to use molecular selection in large-scale breeding programs, it is necessary to introduce automated technologies.

**Figure 3:**
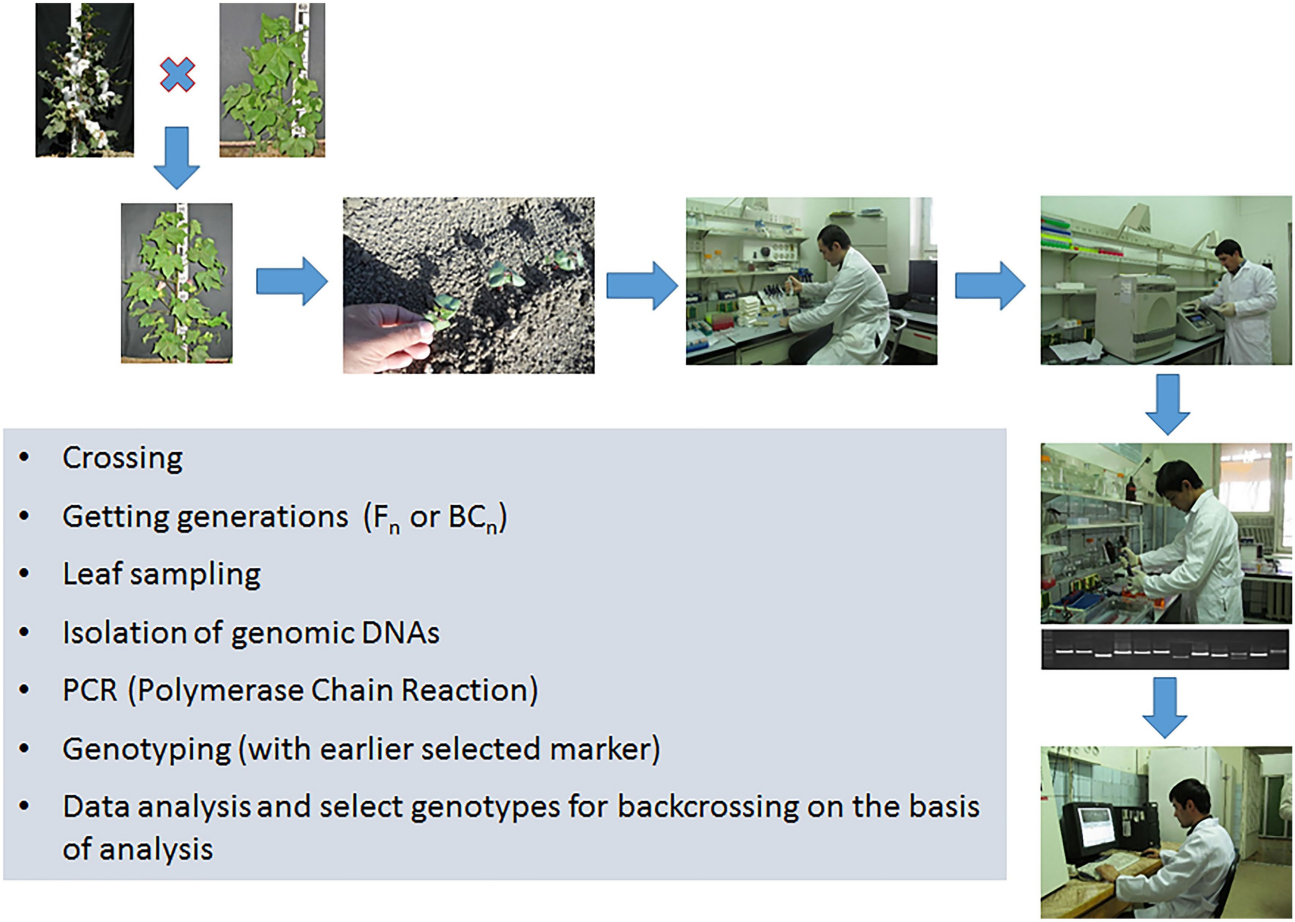
The main procedures of marker-assisted selection technology.

Thus, the basic principle of MAS technology is to identify a tight linkage between the marker and the gene controlling the trait, and subsequently using this association for practical purposes to create new varieties and breeding lines. After the association between the marker and trait has been identified, the creation of new genotypes is carried out using traditional breeding methods, such as hybridization, backcrossing, self-pollination, and selection (Figure 3). Because of using MAS technology, a breeder can get rid of the problem of transfer undesirable genes from the donor, which often occurs during the crossing.

Marker-assisted selection (MAS) technology refers to any form of molecular selection that uses genetic markers to crop improvement (Choudhary et al., 2008; Sebastian et al., 2012). Basically, depending on the goal of the research, MAS is used for the following tasks: (i) Evaluation of the purity and identity of the varietal material and the assessment of the genetic diversity of modern varieties (Williams et al., 1993; Sonah et al., 2011), (ii) Introgression of genes/QTL loci in various MAS schemes (Mackay, 2001; Hospital, 2009), and (iii) Combining several genes/loci of QTL donor lines into one genotype and, thus, the creation of new lines that have several useful traits (Visscher et al., 1996; Hospital, 2009).

In order to effectively use molecular breeding and DNA markers, several strategies for MAS technology have been proposed. However, four main breeding schemes are widely used for crop improvement in practice: (i) Marker-assisted Backcross Selection (MABS), (ii) Marker-assisted Recurrent Selection (MARS), (iii) Marker-assisted Gene Pyramiding (MAGP), and (iv) Genomic Selection (GS; Frisch et al., 1999; Ribaut and Betrán, 1999; Witcombe and Hash, 2000; Collard and Mackill, 2008; Ribaut et al., 2010; Hayes et al., 2013; Varshney et al., 2013; Jiang, 2015; Gokidi et al., 2016; Xu et al., 2017b). All these strategies of molecular selection can be defined as the use of molecular genetic markers, in combination with information of linkage maps and sequenced genomes, to improve the desired traits in plants based on genetic analysis. Among all these schemes, genomic selection is the most popular and widespread method. A relatively new direction, but already a very active area of research in plant and animal breeding – genomic selection, also called Genome-Wide Selection, opens up new exciting prospects for the development of molecular selection for crop improvement (Hayes et al., 2013; Varshney et al., 2013).

### MAS-Based Approaches

Several advanced molecular breeding approaches are used in the creation of crop variety, such as marker-assisted backcrossing (MABC), marker-assisted gene pyramiding, marker-assisted recurrent selection (MARS), and genomic selection (GS). These approaches can help accelerate breeding processes with the early and direct selection of desirable individual plants in the DNA level which are resulting time and resource savings.

#### Marker-Assisted Backcross Selection

The backcross method has been widely used in conventional breeding since the beginning of the last century for the introgression of one or more genes from a donor to an elite variety (Collard and Mackill, 2008). However, the use of DNA markers in backcross programs in combination with phenotypic selection significantly accelerates the production of breeding material (Frisch et al., 1999; Xu et al., 2017b).

**Figure 4:**
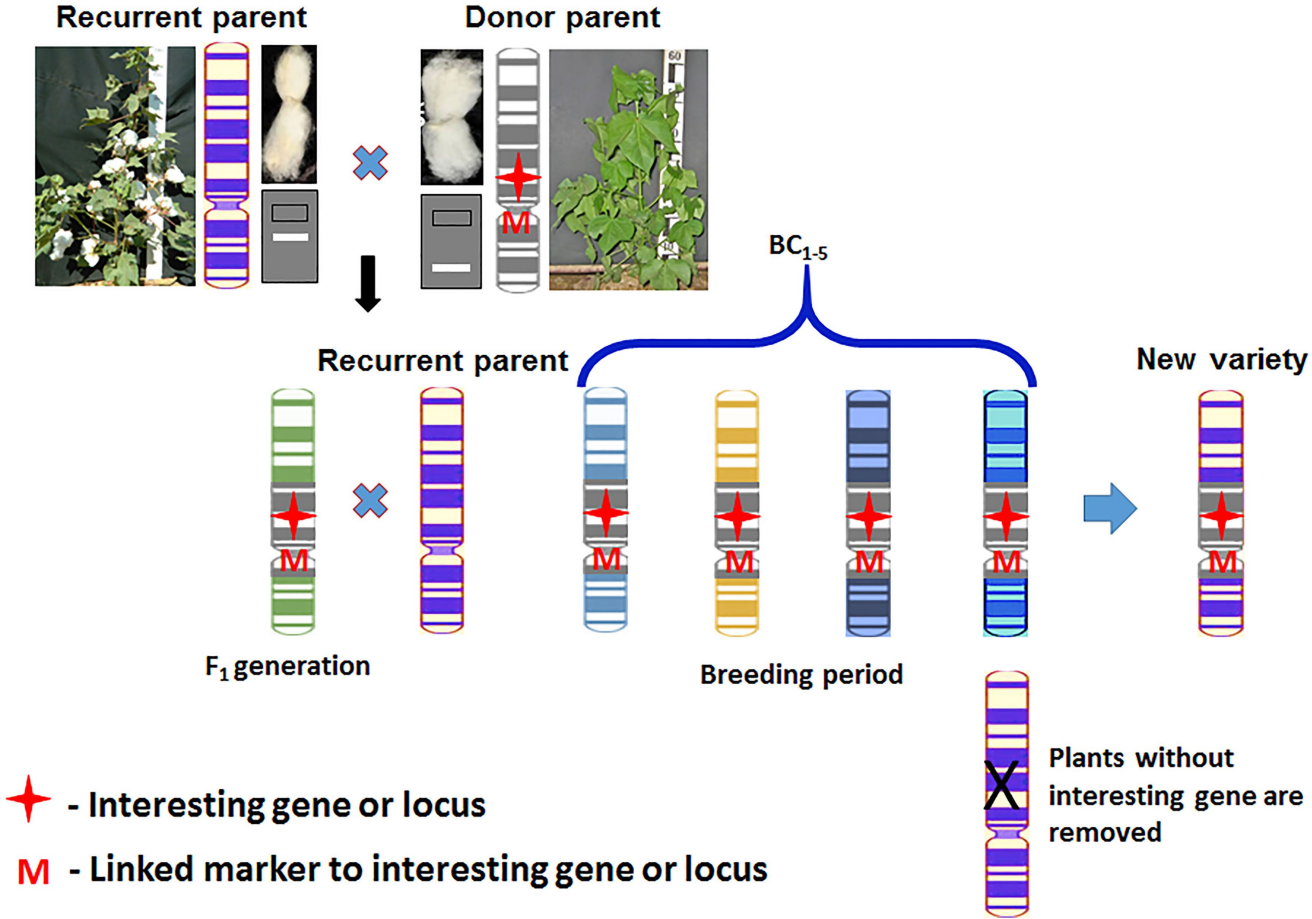
The scheme of marker-assisted backcross selection.

Marker-assisted backcross selection (MABS) is one of the simplest and most promising approaches of MAS technology (Acquaah, 2012). The main goal of MABS is to apply markers to select for target QTL, minimize the length of the donor genome segment containing a target QTL, and accelerate the recovery of the recurrent parent genome (Figure 4). According to Holland (2004), the method has three main levels of breeding, in which markers may be used in backcross selection (Holland, 2004): (i) Foreground selection or selecting of target loci, (ii) recombinant selection or selecting backcross progeny with the target gene, and (iii) background selection or selecting backcross progeny with background markers. These three levels are used in one or another combination in backcross breeding programs for gene introgression.

It is known that for the introgression of one dominant gene it is necessary to carry out at least six backcrosses (Acquaah, 2012), so that in the end the content of the genome of the recurrent parent would be 99% only in theory. With conventional backcrossing, it takes a minimum of five to six generations. The use of DNA markers allows reducing the number of required backcrossing to four and reducing the amount of genetic material transferred with the “target” locus.

MABS has been applied in several important crops, including maize, rice, wheat, barley, common bean, soybean, pear millet, potato, and tomato. For example, the integration of the *Bt* transgene into diverse corn genetic backgrounds has been obtained by using this approach of MAS in maize (Gassmann et al., 2011). MABS strategy was used for rice improvement (Bishwas et al., 2016; Das et al., 2017). According to Rambabu et al. (2015), leaf blast resistance gene was introgressed into variety “Swarna” (Rambabu et al., 2015). In addition, MABS has been used for the effective introgression of favorable alleles from the wild germplasm into elite cultivars; MABS has been used in other crops.

In turn, Li et al. (2013a) based on the results of such research, as well as using the MABS approach, were able to successfully introgress wilt resistance QTL from *G. barbadense* to *G. hirsutum* (Li et al., 2013a). Also, MABS has been initiated in Uzbekistan to improve important fiber traits of cotton. Association mapping has been applied for the identification of QTLs associated with fiber quality, and the selection of donor lines with superior quality using diverse sets of Uzbek cotton germplasm (Abdurakhmonov et al., 2008, 2009). Twenty-six germplasm accessions, as donor lines and more than 10 varieties, as the recipient parents were selected for QTL mobilization through MABS. As a result, new varieties “Ravnaq-1” and “Ravnaq-2” were developed (Darmanov et al., 2015). Both varieties possess higher fiber strength and improved length. “Ravnaq-1” has improved fiber strength (37 g/tex) and staple length (38 mm) compared to its recurrent parent “Andijan-35” which has 32 g/tex fiber strength and 35 mm staple length.

#### Marker-Assisted Recurrent Selection

According to Ribaut et al. (2010), the goal of the marker-assisted recurrent selection (MARS) scheme is the identification and selection of several regions on the genome engaged in the expression of complex quantitative traits to assemble within a single cross or across related populations (Ribaut et al., 2010). Utilization of markers to pyramid for multiple genes or QTLs is more difficult and in this situation, the recurrent selection is a potential approach for the improvement of polygenic traits (Ceballos et al., 2015). As noted by Jiang (2013), the MARS strategy of MAS selection performs genotypic selection and intercrossing in the same crop season for one breeding cycle (Jiang, 2013).

MARS program has been successfully applied to improve important agronomic traits in maize. As described by Beyene et al. (2016), this strategy showed excellent results than the conventional selection in the studies to develop improved drought tolerance germplasm (Beyene et al., 2016). Recently, another research group under the maize improvement program has improved grain yield based on a biparental population using the SNP marker system in MARS (Bankole et al., 2017). However, very little information is available on the practical application of the MAS strategy to improve cotton. According to Yi et al. (2004), cotton bollworm (*Helicoverpa armigera*) resistance of *G. hirsutum* has been increased using MARS (Yi et al., 2004).

#### Marker-Assisted Gene Pyramiding

To create new varieties, using traditional breeding techniques is time consuming, labor-intensive, and can be costly (Moreau et al., 2004). In particular, it is difficult to develop a large number of populations, advancing the hybrids up to F9/F10 generations, the difficulty of the selection processes due to the negative effects of environmental factors on the appearance of morpho-biological traits. In most cases, it is necessary to wait until the last stage of plant ontogenesis to make a selection according to the trait of interest and complexity of combining significant genes in a single genotype (Gupta and Varshney, 2005). In such cases, the selection process has been proven to last for 20–25 years in practice.

Watson and Singh (1952) were first introduced Gene pyramiding conception. According to Allard (1999), pyramiding multiple genes is achieved by crossing parental lines with complementary desirable genes and selecting the desired recombinants from among the progeny population. MAS-based gene pyramiding (MAGP) method is combining at the same time multiple genes/QTLs together into a single genotype using several trait-associated DNA markers. Since the development of this technology, a unique chance has appeared not only to speed up the selection process and reduce costs but also to direct efforts to create varieties with multilateral resistance through gene pyramiding technology. Using traditional breeding methods, it is extremely difficult or impossible to implement this process in the early generations (Sheikh et al., 2017). Nowadays, this technology is considered also as the main acceptable strategy for developing new varieties of crops.

MAGP application had been reported in cotton, wheat, rice, tomato and pepper, etc. The most widespread application of the gene pyramiding method has been for combining multiple disease resistance genes in order to develop durable disease resistance (Ashkani et al., 2015). In recent years, reports have appeared on the application of this technology in cotton. Researchers used the MAGP strategy to combine the major QTL traits of fiber quality and wilt resistance from different donors into one genotype in several commercial cotton varieties in order not only to expand the genetic base of the developed MAS lines but also to ensure their genetic stability. Guo et al. (2005) successfully introduced the effect of pyramiding QTLs for strong fiber strength and transgene cryIA in cotton (Guo et al., 2005). They developed insect-resistant and high-yielding new cotton variety with superior fiber quality.

#### Genomic Selection

Full-genomic sequencing has become available thanks to the development of modern sequencing platforms – next-generation sequencing (NGS) technology. The advantage is the analysis of a large number of markers and the possibility of identifying new genetic variants. NGS technology has made it possible to speed up and cheapen the determination of the complete genome sequence of organisms (Pareek et al., 2011; Zhang et al., 2011). It becomes possible to simultaneously evaluate thousands of genes in organisms, tissues, and cells (sequencing of transcriptomes) and analyze the regulation of their activity. To date, about 300 genomes of different plant species have been sequenced, and this number is increasing each year. The introduction of methods of high-performance genotyping of agricultural organisms opened the way for the application of a new method of selection.

Genomic selection (GS) is considered a novel strategy of MAS for plant breeding, based on the analysis of a large number of DNA markers evenly distributed throughout the genome. The term “genomic selection” was first introduced by Haley and Visscher in 1998 (Haley and Visscher, 1998). Three years later, Meuwissen et al. (2001) developed and presented a GS methodology, as progress in MAS technology for the study of quantitative traits (Meuwissen et al., 2001). In plant breeding, GS has become more productive due to a large number of single-nucleotide polymorphisms (SNPs) detected by sequencing the crop genome. Currently, the full-genome SNP chips have been developed for several types of crops for automatic analysis of DNA polymorphism. According to Meuwissen et al. (2001), the genomic selection also proposes the prediction model based on the genotypic and phenotypic data of the reference population. The reference population is used to receive genomic estimated breeding values (GEBVs) for all individuals of a breeding population based on their genomic profile.

Especially with the advent of the GS method, significant changes have occurred in the evaluation of breeding value in world livestock breeding (Jonas and de Koning, 2015). The accumulation of fundamental knowledge in these areas allowed sequencing the genomes of the main types of agricultural animals – cattle, pigs, and sheep, and carrying out the genotyping of animals by DNA markers. It should be noted that the greatest success in the practical application of GS was noted for Holstein dairy cattle. Naturally, the development of GS methods brought a lot of success also in plant breeding (Wang et al., 2018). An example is a recently published work of Heffner et al. (2010), in which the genetic gain of the GS method in maize breeding is higher than that of its pedigree MAS technology (Heffner et al., 2010). Xu et al. (2014) also confirm that the genomic prediction served to select potential hybrids from recombinant inbred lines (RIL) of rice (Xu et al., 2014). Daetwyler et al. (2014) in their research on wheat rust resistance, applied genomic best linear unbiased prediction (GBLUP), and a Bayesian regression method to predict resistance to leaf, stem, and strip rust (Daetwyler et al., 2014). Gapare et al. (2018) accommodating genotype x environment interaction (GxE) based on a population of 215 breeding lines of tetraploid cotton *G. hirsutum* identified potential breeding lines for fiber length and strength (Gapare et al., 2018). Hulse-Kemp et al. (2015) have developed the CottonSNP63K intraspecific SNPs for use within the Upland cotton (*Gossypium hirsutum* L.) cultivars and interspecific SNPs for use with crosses several cotton species with *G. hirsutum* L. (Hulse-Kemp et al., 2015). Wang et al. (2016) have used CottonSNP70K Chip to detect SNP in four salt tolerance and four salt-sensitive cotton varieties. SNP variation of the same seedling pre- and after-salt stress in different varieties was screened and polymorphic SNP and SNP related to salt tolerance were obtained (Wang et al., 2016). As well, Cai et al. (2017) developed a CottonSNP80K array that plays an important role in germplasm genotyping, variety verification, functional genomics studies, and molecular breeding in cotton by selecting from the re-sequencing data of 100 cotton cultivars (Cai et al., 2017). Above mentioned, SNP arrays are valuable new resources for molecular breeding approaches, such as marker-assisted selection (MAS), marker-assisted gene pyramiding (MAGP), and genomic selection (GS).

Thus, GS is a powerful tool for use in the molecular breeding of crops and is more efficient than MAS for improving complex traits with low heritability (Jannink et al., 2010). This method allows breeders to select new breeding material based on genetic potential. That is, the best hybrid of the breeding population can be selected only on the basis of a simple DNA test instead of waiting for 2–3 years of field data. GS also improves the options for selecting several traits at the same time. The major obstacle for the wide dissemination of this method in the selection of crops is the presence of one of the key stages to analyze SNPs, more precisely the high cost of genotyping.

## Future Perspectives

Genetic diversity is essential to the genetic progress of cotton breeding. The level of genetic diversity is low in *G. hirsutum*, especially among agriculturally elite types, as revealed in many previous studies (Gutiérrez et al., 2002; Abdurakhmonov et al., 2009, 2012). Chromosome substitution lines (CSL) have been used to overcome the problem of interspecific introgression using conventional breeding methods (Saha et al., 2013, 2017; Jenkins et al., 2017a,b). Chinese scientists used a different approach than CSL to introgress alleles from *G. barbadense* into *G. hirsutum*, where they have developed chromosome segment introgression lines using TM-1, *G. hirsutum*, as the recipient and a high-quality *G. barbadense* line “Hai7124” as the donor (Wang et al., 2012). After four cycles of the MAS breeding program using markers specific to the donor line Hai7124, they developed 174 lines containing 298 introgressed segments with 86 lines having single introgressed segments. “The total length of introgressed segments covered 2948.7 cM with an average segment length of 16.7 cM and represented 83.3% of the tetraploid cotton genome” (Wang et al., 2012). These lines were used in the genetic dissection of the complex fiber quality traits, with 43 additive QTLs and six epistatic QTLs associated with fiber quality traits in a molecular map (Wang et al., 2012). Jenkins et al. (2018) crossed 18 *G. barbadense* CSL to three Upland cotton cultivars and developed a random mated population for the cotton breeding program (Jenkins et al., 2018). After five cycles of random mating using a mixer of pollens from individual CSL followed by one generation of self-pollination to increase the seed supply to develop the random mated population with improved genetic diversity. They used 139 *G. barbadense* chromosome-specific SSR markers to assess a random sample of 96 plants for introgression. They detected 121 of 139 marker loci among the 96 plants. The number of *G. barbadense* alleles ranged from 10 to 28 in each individual plant. They also discovered that the individual plants among the 96 plants had marker loci from 6 to 14 different chromosomes or chromosome arms. However, results on the identity by descent showed little relatedness among plants and no population structure was indicated by a heat map. Using CSL, they were able to develop a mostly Upland random mated population with considerable introgression of *G. barbadense* alleles which would be useful for the cotton breeding program.

Recently, very cost-effective high-throughput sequencing technologies open up a new paradigm in the molecular cotton breeding programs using RNA-seq technologies. High-throughput sequencing technologies are used for RNA-seq experiments to generate cDNA sequences derived from the total RNA molecules followed by library construction and massively parallel deep sequencing to quantify the abundance level of relative changes of the individual transcripts at a specific stage of development or under specific treatment conditions. The application of the RNA-seq tool to associate changes in gene expression from high-throughput results of transcriptomics with low background noise to associate with important traits shows great potential in a future cotton breeding program. Recently, Naoumkina et al. (2019) used RNA-seq analysis in a GWA study in a MAGIC population. RNA-seq analysis of the longest and shortest fiber length RILs from D-11ref and D-11alt populations detected 949 significantly differentially expressed genes (DEGs; Naoumkina et al., 2019). Gene set enrichment analysis identified that different functional categories of genes were overrepresented during fiber elongation between four selected RILs. They discovered that 12 genes possessing non-synonymous SNPs were significantly associated with the fiber length. They also detected that in close proximity to fiber length QTL on chromosome D11, an auxin-responsive GH3 gene with a significantly downregulated expression level in one of the longest fiber length RILs suggesting that it could play a role in the regulation of fiber-cell elongation.

Transcriptome-wide association studies (TWAS) are a powerful strategy that integrate GWAS and gene expression datasets for identification of gene-trait associations (Wainberg et al., 2019; Bhattacharya et al., 2021). Recently, Li et al. (2020b) performed a fiber transcriptome analysis by sequencing of natural *G. hirsutum* population with 251 accessions and identified 15,330 expression quantitative trait loci (eQTL) associated with 9,282 genes (Li et al., 2020b). Analysis of eQTL and GWAS data uncovered molecular regulation of cotton fiber development and revealed the genetic basis of cell wall synthesis during fiber-cell elongation.

The results of above mentioned studies using the RNA-seq and TWAS tools provided an insight into the molecular aspects of genetic variation and fiber development as well recommended the potential sources for MAS and genetic manipulation technologies, such as CRISPR, in the future cotton improvement programs.

## Conclusion

Cotton is the most important source of natural fiber worldwide. The negative impacts of the natural environment like water scarcity, soil salinization, diverse insect pests, and diseases cause serious damage to cotton productivity and fiber quality. It is imperative to create new cultivars with high yield, superior fiber quality, and resistance to the biotic and abiotic stresses through the use of diverse germplasm resources including wild cotton species and utilize high-throughput technologies. The use of genetic diversity of cotton species and populations in genetic mapping of quantitative traits allows to identify genome-wide informative DNA markers or genes and to determine potential breeding donors with desirable traits. Marker-assisted selection-based molecular breeding approaches could be helpful in pyramiding multiple genes/QTLs linked with resistance, quality, and yield components into a single genotype (Dormatey et al., 2020). Progress in this area will be further increased by taking the information generated through “omics” studies (Boopathi, 2020). Furthermore, as stated above, involving innovative approaches, combining diverse resources and enhance the capacities for increasing marker-assisted selection in cotton ultimately result in developing cotton cultivars with improved quality and productivity.

## Author Contributions

FK, OT, and DE wrote the first draft of manuscript. SS, JY, and IA provided the critical comments and edited the manuscript. All authors listed have made substantial, direct, and intellectual contributions to the work, and approved the manuscript.

### Conflict of Interest

The authors declare that the research was conducted in the absence of any commercial or financial relationships that could be construed as a potential conflict of interest.

### Publisher’s Note

All claims expressed in this article are solely those of the authors and do not necessarily represent those of their affiliated organizations, or those of the publisher, the editors and the reviewers. Any product that may be evaluated in this article, or claim that may be made by its manufacturer, is not guaranteed or endorsed by the publisher.

## Footnotes

^1^ http://www2.cottonqtldb.org
